# The effect of preoperative short-term octreotide treatment to surgery in thyrotropin-secreting pituitary adenomas: a retrospective cohort study

**DOI:** 10.1186/s12902-023-01398-z

**Published:** 2023-08-17

**Authors:** Runsheng Zhao, Kaiyu Fan, Weiqing Wan

**Affiliations:** 1 Department of Neurosurgery, Beijing Tiantan Hospital, Capital Medical University, Beijing, China; 2 China National Clinical Research Center for Neurological Diseases, Beijing, China

**Keywords:** Thyrotropin-secreting pituitary adenomas, Octreotide, GTR, Biochemical remission rate

## Abstract

**Background:**

To prevent thyroid storm and ensure surgical safety, it is imperative to regulate excessive thyroid hormone levels in patients with thyrotropin-secreting pituitary adenomas (TSHoma) prior to surgery. Somatostatin analogues (SSAs), such as octreotide, have showed efficacy in shrinking tumors, which may facilitate surgical resection. This retrospective study aimed to investigate the effect of shortterm preoperative octreotide treatment on the surgical outcome of TSHoma.

**Methods:**

A total of 65 TSHoma patients from January 2010 to July 2019 were included in the study. Of these,41 patients received short-term preoperative octreotide (Sandostatin, intermittent subcutaneous injection) treatment and all patients subsequently underwent surgery. The following data were recorded: clinical manifestations, laboratory examinations, sellar region MRI, postoperative pathological and electron microscopy data, intraoperative situation, and follow-up (> 3 months) regarding hormone levels and tumor recurrence.

**Results:**

There was no significant difference in the consistency and blood supply of the tumor between patients who received short-term preoperative octreotide treatment and those who did not. Additionally, preoperative short-term octreotide treatment (median of 10 days with a range of 6–18 days) did not significantly improve the rates of gross total resection (GTR) or biochemical remission. Moreover, electron microscopy revealed subcellular level impairments and cell apoptotic in the octreotide treated TSHoma specimens.

**Conclusion:**

Preoperative octreotide treatment for the purpose of reducing excessive thyroid hormones may not enhance surgical outcomes, and the duration of octreotide treatment needs to be extended to fully benefit from the tumor-shrinking effects of SSAs.

**Supplementary Information:**

The online version contains supplementary material available at 10.1186/s12902-023-01398-z.

## Introduction

Thyrotropin-secreting pituitary adenomas (TSHoma) is a rare subtype of pituitary adenomas accounting for 0.5-3.0% of all functional pituitary adenoma [1–3]. Primary clinical manifestations of TSHoma are hyperthyroidism and neurologic manifestations from mass effect [2, 4]. Surgical resection is the primary therapeutical approach for TSHoma. In cases where surgery is either not recommended or declined, radiotherapy should be considered as an alternative option. Medication such as antithyroid drugs or somatostatin analogues can only restore euthyroidism and control symptom [5].

Excessive thyroid hormones caused by TSHoma can increase the risk of perioperative thyroid storm. Therefore, it is imperative to reduce high hormone levels prior to surgery [6]. Somatostatin analogues (SSAs) were originally used to treat growth hormone pituitary adenomas (GHoma) by binding to somatostatin receptors (SSTRs). Since SSTRs are also expressed on TSHoma cells, SSAs can also be used to inhibit TSH secretion [7–9]. A guideline from the European Thyroid Association recommends administering antithyroid drugs (ATD, such as methimazole or propylthiouracil) or SSAs to decrease thyroid hormone levels before surgery [10].

Some studies have found that SSAs can normalize thyroid hormone levels in approximately 80% of patients and induced tumor shrinkage in about 45% of cases [11]. Currently, there is no consensus on the preoperative SSAs treatment for TSHoma, and the preoperative treatment duration depends on the time it takes to normalize thyroid hormone levels. Except for normalizing hormone levels, short-term treatment may cause changes in the tumor consistency or even tumor size, which could have a positive effect on surgical outcomes. Fukuhara had mentioned the effect of preoperative SSAs on surgery in his trial [12], but further elaboration was insufficient. It is uncertain whether short-term preoperative treatment has any effect on surgery. The aim of this study is to evaluate the effect of preoperative short-term octreotide treatment on surgery.

## Materials and methods

### Patients and diagnostic criteria

We retrospectively collected data of 65 consecutive patients with TSHoma diagnosed from January 2010 to July 2019 at Beijing Tiantan Hospital. Diagnostic criteria: (1) Presence of such clinical manifestations as thyrotoxicosis and/or symptoms and signs attributed to the intracranial mass effect; (2) Significant increase of free thyroid hormone levels and normal/high TSH at the same time; (3) Positive SSAs suppression test: The release of TSH is inhibited after the administration of somatostatin analogues (SSAs); (4) MRI results indicate pituitary adenoma; (5) Histological examination by immunohistochemistry proved positive for TSH, with or without positive for GH, PRL, FSH, LH, and ACTH. Due to medical insurance not covering SSAs treatment for TSHoma and the availability of short-term ATDs for preoperative preparation, some patients (15 cases) rejected the preoperative octreotide treatment and opt for ATDs treatment instead. Additionally, some patients (9 cases) with slightly elevated FT3 and FT4 and mild hyperthyroidism were able to tolerate surgery after preoperative evaluation. The patients were divided into two groups(41vs.24) based on whether they received preoperative octreotide treatment (Sandostatin, intermittent subcutaneous injection).

### Hormone examination

Thyroid stimulating hormone (TSH, 0.49-4.91uIu/ml), adrenocorticotropic hormone (ACTH, 0.0-46.0pg/ml), growth hormone(GH 0.0-3.0ng/ml), luteinizing hormone (LH, 0.8–7.6 uIu/ml) / follicle stimulating hormone (FSH, 0.7-11.1uIu/ml) and prolactin (PRL 2.5-17.0ng/ml), as well as the corresponding target gland (thyroid, adrenal and gonadal) hormones, including total triiodothyronine (TT3, 1.01-2.48nmol/l), free triiodothyronine (FT3, 3.28-6.47pmol/l), total thyroxine (TT4, 69.97-152.52 nmol/l), free thyroxine (FT4, 7.64-16.03pmol/l), cortisol, insulin-like growth factor 1 (IGF-1, 45-210ng/ml), testosterone, progesterone and estradiol levels were measured using a microparticle chemiluminescence immunoassay (Abbott Ireland Diagnostics Division, Longford, Ireland).

### Imaging examination

All the patients underwent a contrast enhanced MRI examination of the sellar region to identify the size, shape, location, and peripheral adjacency relationships. Based on the largest diameter, the tumor size was classified as microadenoma(≤ 10 mm), macroadenoma(10-40 mm), or giant adenoma(≥ 40 mm). Tumor volume was estimated by multiplying the length, width, and height and then multiplying the result by 0.523.

### Intervention

1. Medication: The subcutaneous injection of 100ug of Octreotide (Sandostatin, Novartis Pharma) was administered at 8-hour intervals. If there were adverse reactions such as nausea, vomiting, abdominal pain, or diarrhea, the dosage was decreased to 100ug twice or once daily the following day. If the adverse reactions persisted, octreotide treatment was discontinued. After injection, thyroid hormones and TSH were monitored in the morning daily until FT4 returned or close to normal. The number of injections, adverse reactions, total dose were recorded. The endpoint of medical treatment was the normalization of free thyroid hormone concentrations. For some patients who were not sensitive to octreotide, the goal was to control hormone levels to a lower level.
2. Surgery: All patients underwent surgery at the Neurosurgery Department of Beijing Tiantan Hospital. Resected tumors were sent to the Pathology Department for routine pathological examinations and immunohistochemistry analysis. The surgical approach was determined based on the characteristics of the tumor, such as transsphenoidal or craniotomy. After surgery, the surgeon recorded the duration of the operation, surgical bleeding, tumor consistency, tumor resection rate, and surgical complications. The resection rate was confirmed as gross total resection (GTR), subtotal resection (STR), or partial tumor resection (PTR) based on postoperative MRI.

### Electron microscopy

Electron microscope examinations were performed on specimens from TSHoma patients who received octreotide treatment. The 1-mm-thick sections were double-fixed in 2.5% glutaraldehyde (by volume) and 2% formaldehyde solution for 2 h at 4℃, and then fixed in 1% osmium tetroxide, dehydrated in an ethanol gradient, and embedded in labeled embedding molds using fresh EMbed-812, and polymerized. The section blocks were confirmed and cut into 60-nm sections, stained in uranyl acetate and lead citrate, and their specific ultrastructure features were observed under a Hitachi H-7650 (120 kV) transmission electron microscope. One set of electron microscopy data was randomly selected from one TSHoma patient and eight patients who received preoperative octreotide treatment.

### Postoperative pituitary function assessment and criterion of biochemical remission

Blood samples were taken from patients in 8–12 h and 3 days after surgery to measure the serum hormone levels. The results were used to evaluate the pituitary and its target gland function and assess the intraoperative damage to the pituitary. Thyroid hormones and TSH were measured more than 3 months postoperatively to determine if biochemical remission had been achieved. The criterion of biochemical remission of TSHoma is defined as disappearance of hyperthyroidism symptom, normal or decreased TSH level, and normal free thyroid hormone levels.

### Statistical analysis

SPSS 25.0 (SPSS Inc., Chicago, IL) statistics software was used for the analysis. Measurement data were expressed as mean ± standard deviation if they were normally distributed, while data that were not normally distributed were expressed as median and interquartile range. Normal distribution data for the comparison of pituitary-thyroid axis hormone changes were tested using paired samples t-tests, while data that were not normally distributed were analyzed using rank sum tests. Categorical variables were expressed as case numbers and percentages. Categorical variables were compared using the χ2 test or Fisher’s exact test. A p value < 0.05 was considered statistically significant.

## Results

### Characteristics of the patients

The total number of patients who received the preoperative short-term octreotide treatment was 41(63%), while 24 (37%) patients did not receive such treatment. Table 1 presents the characteristics of the patients, and it shows that age, gender ratio, and tumor size were not significantly different between the two groups. The clinical manifestations exhibited by patients with TSHoma primarily comprise of symptoms of hyperthyroidism and those caused by intracranial mass effect. Some patients in both groups exhibited no obvious neurological symptoms but only symptoms of hyperthyroidism, which led to misdiagnosis as primary hyperthyroidism. Mixed TSHoma secreted TSH accompanied by excessive secretion of GH or PRL, and the proportion of mixed TSHoma was higher in the treatment group (14% vs. 12.5%). Despite higher levels of FT3 and FT4 in the group that received preoperative octreotide treatment, there were no significant differences in free thyroid hormone levels between the two groups (Table 1).

**Table 1:** Clinical characteristic: patients with or without preoperative short-term octreotide treatment

Group		Pre-OCT (n = 41)	Non-pre-OCT (n = 24)	P
M/F	23/18		14/10	0.86
Age(y)	40.37 ± 11.75		40.63 ± 12.48	0.93
Tumor size(mm)				
< 10	4		2	0.97*
10–40	30		18	
> 40	7		4	
No neurological symptoms	25(61.0%)		10(41.7%)	0.13
Mixed TSHoma (GH/PRL)	6(14.6%)		3(12.5%)	0.81
Free T3 (nmol/L)	9.67 ± 3.51		8.31 ± 1.46	0.08
Free T4 (nmol/L)	26.25 ± 6.61		23.23 ± 5.52	0.05
TSH (µIu/ml)	5.63 ± 5.18		5.01 ± 3.05	0.60

Pre-OCT: patients with preoperative octreotide treatment. Non-pre-OCT: patients without preoperative octreotide treatment.

* Fisher’s Exact Test:1 cell (25.0%) has expected count less than 5.

### Preoperative short-term octreotide treatment outcomes

**Fig. 1:**
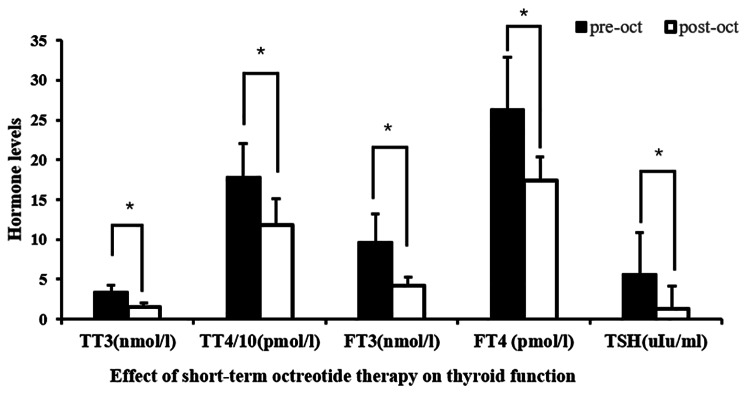
Effect of short-term octreotide treatment on TSH and thyroid hormones levels

The preoperative octreotide treatment lasted between 6 and 18 days, with a median duration of 10 days. The median dose of octreotide administered was 2.2 mg, ranging from 1.2 to 4.5 mg. Following the preoperative treatment, all patients displayed a significant decrease in TSH and thyroid hormone levels (Fig. 1, TT3 [nmol/L]: 3.35 ± 0.92 to 1.53 ± 0.46; TT4 [pmol/L]: 178.22 ± 42.50 to 118.28 ± 32.45; FT3 [nmol/L]: 9.67 ± 3.51 to 4.22 ± 1.06; FT4 [pmol/L]: 26.25 ± 6.61 to 17.41 ± 2.96; TSH [µU/ml]: 5.63 ± 5.18 to 1.33 ± 2.84; more data shown in **Additional file 2**). In 70% (29/41) of patients, FT4 and FT3 concentrations returned to normal levels. The maximum values of FT3 and FT4 concentrations were 6.37 nmol/L and 25.67 pmol/L, respectively, after treatment. One patient experienced mild diarrhea after injection, but the symptoms disappeared 2 days after the dosage was reduced to 100 µg qd. No patients discontinued treatment due to intolerable adverse reactions.

**Fig. 2:**
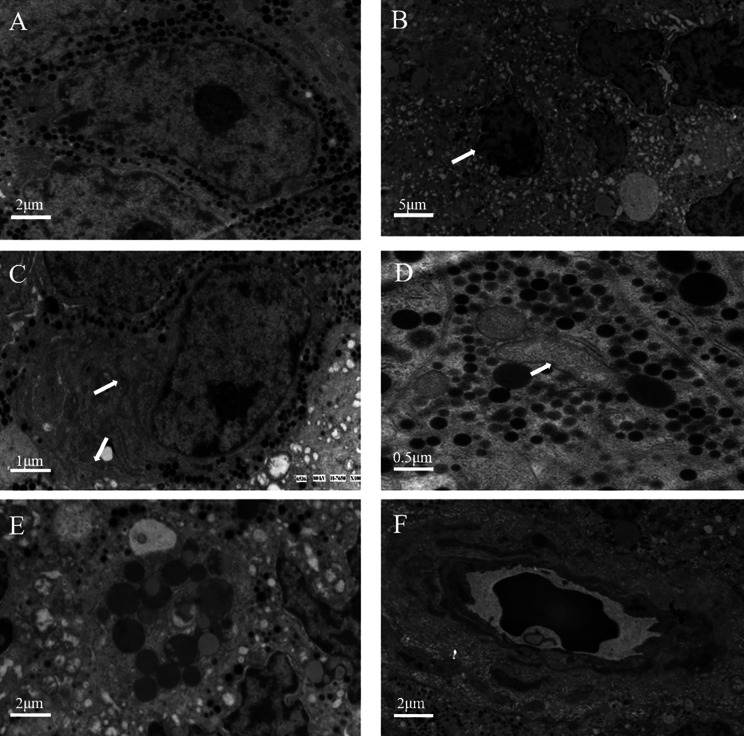
The ultrastructure of tumor cells under electron microscopy between patients without or with preoperative treatment

To further verify the effect of octreotide, the resected tumor tissues were analyzed using electron microscopy. Compared with the specimens from patients who did not receive preoperative treatment (Fig. 2A), those from patients who underwent octreotide treatment showed various changes in the subcellular structure of the tumor. These changes included mitochondrial swelling, endoplasmic reticulum dilatation and vacuolization, membrane dissolution and fracture, blurring of the nucleus and organelle, and cell disintegration (Fig. 2B-D). Characteristic apoptotic morphological changes were observed in TSHoma cells (Fig. 2E, F).

### Surgical outcome and biochemical remission rate

Based on intraoperative observation and analysis, there were no significant differences in tumor consistency (P = 0.92), tumor blood supply(P = 0.58) and intraoperative blood loss (0.33) between the two groups. (Table 2) In our patients, the tumor volume (10.97 ± 14.86cm^3^ vs. 9.08 ± 13.88 cm^3^,P = 0.61) and cavernous sinus invasion rate(P = 0.19), which are the preoperative factors that could impact surgical resection rate, showed no significant differences between the two groups. Moreover, preoperative short-term octreotide treatment had no significant effect on tumor resection rate(P = 0.34) or surgical complications, as detailed in Table 2.

**Table 2:** Effects of short-term octreotide treatment on surgical outcomes

Group	Pre-OCT (n = 41)	Non-pre-OCT (n = 24)	p
tumor volume(ml)	10.97 ± 14.86	9.08 ± 13.88	0.61
CS invasion	17/41	14/24	0.19
tumor consistency			
soft	20	12	0.92
tough	21	12	
blood supply			
medium	30	16	0.58
rich	11	8	
intraoperative blood loss (ml)	274.63 ± 346.50	197.08 ± 230.66	0.33
surgical resection rate			
GTR	23	21	0.34*
STR	16	3	
PTR	2	0	
surgical complications			
infection	4	3	0.70
diabetes insipidus	6	8	0.08
CSF leakage	2	0	0.53
injury of surrounding tissues (Optic nerve, hypothalamus)	2	0	0.53

Pre-OCT: patients with preoperative octreotide treatment. Non-pre-OCT: patients without preoperative octreotide treatment. CS invasion: cavernous sinus invasion; GTR: gross total resection; STR: subtotal tumor resection; PTR: partial tumor resection. *Kruskal Wallis Test.

The postoperative hormone levels at 3 days after surgery were used to assess surgical outcome by evaluating TSH decrease and assess the invasion of normal pituitary tissue by other related hormone decrease. As shown in Table 3, there was no difference between two groups regarding surgical outcomes and invasion of normal pituitary tissue. All patients were followed-up for at least 3 months following surgery, except for one patient who was lost to follow-up in each group, Free thyroid hormone concentrations were normalized in 32 out of 40 (80%) of patients with short-term octreotide treatment compared to 20 out of 23 (86.9%) in patients without octreotide treatment. The biochemical remission rate in the preoperative octreotide treatment group was no significantly difference from those who did not receive preoperative treatment (P = 0.72) (Table 3).

**Table 3:** Postoperative neuroendocrine function (3 days) and biochemical remission (> 3 months)

Group	TSH↓	COR↓	IGF-1↓	PRL↓	FSH/LH↓	biochemical remission (> 3 months)
Pre-OCT	25/41(61.0%)	5/41 (12.2%)	1/41 (2.4%)	18/41 (43.9%)	8/41 (19.5%)	32/40(80%)
Non-pre-OCT	14/24(58.3%)	8/24 (33.3%)	1/24 (4.2%)	9/24 (37.5%)	9/24 (37.5%)	20/23(86.9%)
P	> 0.05					

Pre-OCT: patients with preoperative octreotide treatment. Non-pre-OCT: patients without preoperative octreotide treatment. TSH: Thyroid stimulating hormone. COR: cortisol. IGF-1: insulin-like growth factor 1. PRL: prolactin. FSH/LH: luteinizing hormone or follicle stimulating hormone.

## Discussions

The present study was a retrospective analysis of the effects of preoperative octreotide treatment on the surgical outcome and biochemical remission rate of TSHoma patients. Based on intraoperative observation and postoperative MRI, preoperative treatment did not improve the tumor resection rate in TSHoma patients. Additionally, according to the biochemical remission criteria, the postoperative TSH remission rates in patients who received preoperative octreotide treatment (80%) were lower compared to those in patients without preoperative octreotide treatment (86.9%), but the difference was not significant. This study also demonstrated that short-term octreotide treatment can significantly decrease thyroid hormone levels and induce varying degrees of apoptosis of the TSHoma cells.

TSHoma typically presents with a fibrous and hard consistency due to the expression of fibroblast growth factor [13, 14]. Furthermore, there is a high proportion of macroadenomas or tumor extrasellar extension, which may be due to delayed diagnosis and prior ATDs treatment [15]. These tumor characteristics make GTR challenging. In a recent meta-analysis, the GTR rate in TSHoma was found to be slightly lower, with approximately 54% of patients achieving complete resection [16]. Another challenge in the treatment of TSHoma is preoperative management, as patients are required to regulate their free thyroid hormone concentrations to as close to normal levels as possible prior to surgery to enhance surgical safety. SSAs were originally used to control high level of GH in acromegaly patients. SSAs stimulate p27 expression and inhibit the MAPK pathway in pituitary tumors by binding to SSRT2 and SSRT5 [17–19].Studies have found that SSTR2 and SSTR5 also express in TSHoma cells, suggesting that SSAs can be used to treat TSHoma [8, 9]. The secretion of TSH is mediated through the interactions between SSAs and SSRT2, whereas the reduction in tumor size is influenced mainly by the interactions between SSAs and SSRT5 [20, 21].

It has been reported that short-term preoperative octreotide treatment can induce apoptosis in tumor cells, as observed through electron microscopy [9]. In the long-term, TSH secretion suppression rates of about 90% can be achieved, and around 70% of patients can recover normal free thyroid hormone concentrations [22]. Additionally, preoperative SSAs treatment can lead to a reduction in tumor size, with one study reporting a 50% shrinkage rate [23].Given these advantages, Fukuhara had studied the effects of preoperative octreotide on surgery, which showed that short-term use had no significant impact on tumor consistency or surgical cure rate [12]. In our research, we conducted further investigations into the surgical resection rate and the biochemical remission rate, and our findings were consistent with Fukuhara’s findings, but more specific. Our research found preoperative short-term octreotide treatment could not improve the surgical outcomes. and no changes were observed in tumor consistency.

Our research suggests that treatment duration may be a significant factor influencing the outcomes of TSHoma treatment. Currently, the duration of SSAs treatment is determined by the time required for free thyroid hormone levels to return or approach normal levels in the TSHoma patients who are scheduled to undergo surgery. In a phase 3 clinical trial on SSAs treatment for TSHoma patients, most patients became euthyroid within 1 month [24]. In our study, treatment lasting 2 weeks induced low levels of FT4 concentrations in most patients. However, differences in treatment duration may result in varying extents of tumor shrinkage, and a greater reduction in tumor size may have a more positive impact on the surgical outcomes. Several studies have suggested that preoperative SSAs treatment could improve the surgical recovery rate in GHoma patients, especially in those with macroadenomas. In the literature, the duration of SSAs treatment generally ranged from 3 to 6 months [25]. Fukuhara’s study found that tumor shrinkage was observed in 61% of patients after a median of 37 days. It took longer for the tumor to shrink than for the FT4 normalization, which was observed in 84% of patients after a median 20 days [12]. A longer treatment duration is often necessary to reduce tumor size and achieve a favorable effect on surgery. Long-acting somatostatin analogs (SSAs) may be more effective in shrinking tumors and can be administered every 21 or 28 days. However, the use of long-acting SSAs must be considered in relation to their effects on euthyroidism and the patient’s willingness to undergo surgery.

The novelty of the present study lies in its focus on the effects of preoperative short-term octreotide treatment on surgical outcomes. Our study confirmed that short-term octreotide treatment aimed at controlling excessive thyroid hormone secretion is not sufficient to impact surgical outcomes. The purpose of SSAs treatment should not only be to control excessive thyroid hormone levels but also to reduce tumor size. Prolonged SSAs treatment duration after thyroid hormone levels have normalized may result in tumor shrinkage in more patients, particularly those with macroadenomas or tough consistency.

The present study has some inherent limitations due to its retrospective nature, such as selection bias and information bias. Moreover, the most significant limitation of this study was the lack of MRI after preoperative treatment, which prevented us from determining the rate of tumor shrinkage accurately. The lack of objective criteria for tumor consistency, which was judged by surgeons intraoperatively, may have led to inaccurate results. The long-time span of enrollment and the different follow-up times may have resulted in a higher postoperative biochemical remission rate than the actual situation. These limitations can be addressed in future prospective controlled studies. Future studies are determined whether an prolonged preoperative SSAs treatment duration can improve surgical outcomes and to determine the appropriate duration of SSAs treatment.

## Conclusion

TSHoma is a rare subtype of pituitary adenoma that presents challenges for surgical treatment due to its characteristics. Preoperative octreotide treatment can rapidly curb excessive TSH and thyroid hormones secretion and minimize surgical risks. Currently, there is no consensus on the optimal duration of preoperative SSAs treatment for TSHoma patients. While short-term octreotide treatment can restore euthyroid, it does not improve the surgical outcomes. Prolonged SSAs treatment duration may further enhance their ability to shrink tumor size, which can potentially improve the surgical outcomes.

### Electronic supplementary material

Below is the link to the electronic supplementary material.

Supplementary Material 1

Supplementary Material 2

## Data Availability

All data generated or analyzed during this study are included in this published article and its supplementary information files.

## List of abbreviations

TSHoma: thyrotropin-secreting pituitary adenomas
SSAs: somatostatin analogues
GHoma: growth hormone pituitary adenomas
SSTR: somatostatin receptor
OCT: octreotide
ATD: antithyroid drugs
GTR: total resection
STR: subtotal resection
PTR: partial tumor resection
CS: cavernous sinus
